# Tendoscopy-Assisted Flexor Digitorum Longus Transfer and Spring Ligament Synthetic Suture Tape Reconstruction for Flexible Progressing Collapsing Foot Deformity

**DOI:** 10.1016/j.eats.2025.103781

**Published:** 2025-07-30

**Authors:** Donatas Chlebinskas, Ciaran Nolan

**Affiliations:** Sheffield Teaching Hospitals, Sheffield, U.K.

## Abstract

Progressive collapsing foot deformity is a complex deformity that is predisposed to by weakening and elongation of the spring ligament, resulting in peritalar subluxation. A medializing calcaneal osteotomy and flexor digitorum longus tendon transfer are standard surgical procedures for a symptomatic flexible deformity that is recalcitrant to nonoperative treatment. Endoscopic-assisted flexor digitorum longus transfer and a minimally invasive medializing calcaneal osteotomy, allow a fully minimally invasive approach to address flat foot reconstruction. Concerns remain regarding low correction potential, lack of medial longitudinal arch reconstitution, and a lack of improvement of forefoot abduction without surgically addressing the attenuated spring ligament. Spring ligament augmentation with an internal brace provides benefits of high resistance to forefoot lateralization, reliably improved radiographic parameters, and patient-reported outcomes. We describe a tendoscopic-assisted flexor digitorum longus tendon transfer and a spring ligament synthetic suture tape reconstruction technique, used in conjunction with a medializing calcaneal osteotomy. This technique allows a completely minimally invasive approach to flexible progressive collapsing foot deformity reconstruction, with increased resistance to forefoot lateralization and the potential to more reliably improve and preserve radiographic parameters.

Progressive collapsing foot deformity (PCFD) is a complex pathology defined by the collapse of the medial longitudinal arch and a valgus deformity of the hindfoot. Forefoot abduction, forefoot varus, ankle instability, and valgus deformity may develop in the later stages. Each component of the deformity can commence as flexible and progress to rigid and may lead to arthritis of affected joints.^1^

The prevalence of plano-valgus deformity was reported to be 17% among non-Hispanic whites and 34% among African Americans by Dunn et al.^2^ Women are more commonly affected than men, and the median age of presentation is 55 years.^3^ Obesity, hypertension, diabetes, hyperlipidemia, genetic predisposition, and high-impact sports have been identified as contributors to the development of the pathology.^1^ Up to 76% of patients with PCFD have an associated tight gastrocsoleus complex.^4^

Posterior tibial tendon degeneration, elongation, and weakening were traditionally thought to be a key etiopathological component in the development of posterior tibial tendon dysfunction following a landmark paper by Johnson and Strom in 1989.^5^ Later studies have shown that spring ligament weakening and elongation allow peritalar subluxation, potentially initiating the deformity before the posterior tibial tendon is subjected to overload.^6^ The plantar fascia and deltoid ligament complex are also often elongated because of prolonged strain in chronic planovalgus deformity.

Patients with PCFD will initially exhibit medial hindfoot pain and progress to subfibular pain and painful arthritis as the condition worsens.^1^

Medializing calcaneal osteotomy and flexor digitorum longus tendon transfer is a traditional procedure for flexible deformity in cases recalcitrant to nonoperative management, with good to excellent reported outcomes in mid- and long-term cases series.^7^^,^^8^ Concerns remain regarding the lack of medial longitudinal arch reconstitution and no improvement of forefoot abduction with the original technique.

Spring ligament repair can be attempted, but the ligament is often too degenerate for a reliable primary repair and fails early with weightbearing. Spring ligament complex internal brace augmentation has been described by Acevedo and Vora.^9^ Spring ligament internal brace augmentation has shown superior resistance to forefoot lateralization in a cadaveric specimen study and reliably improved radiographic parameters in a case series.^10^^,^^11^

A technical note on endoscopic-assisted flexor digitorum longus (FDL) transfer has been published by Elkaim et al.^12^ and offers an all minimally invasive approach to flatfoot reconstruction. This technique does not address the spring ligament. Endoscopic techniques to repair the spring ligament have been described before, but concern regarding spring ligament fitness for repair indicates limited benefit from such procedures.^13^ We describe a tendoscopic-assisted FDL tendon transfer and spring ligament synthetic suture tape reconstruction technique, permitting an all minimally invasive approach to flexible PCFD reconstruction with increased resistance to forefoot lateralization and the potential to improve and preserve radiographic parameters more reliably.

Indications for this technique include clinically and radiologically confirmed symptomatic flexible PCFD with hindfoot valgus, medial longitudinal arch collapse, a midfoot break at the talonavicular joint, and less than 40% of talonavicular coverage angle.

## Surgical Technique

**Fig 1:**
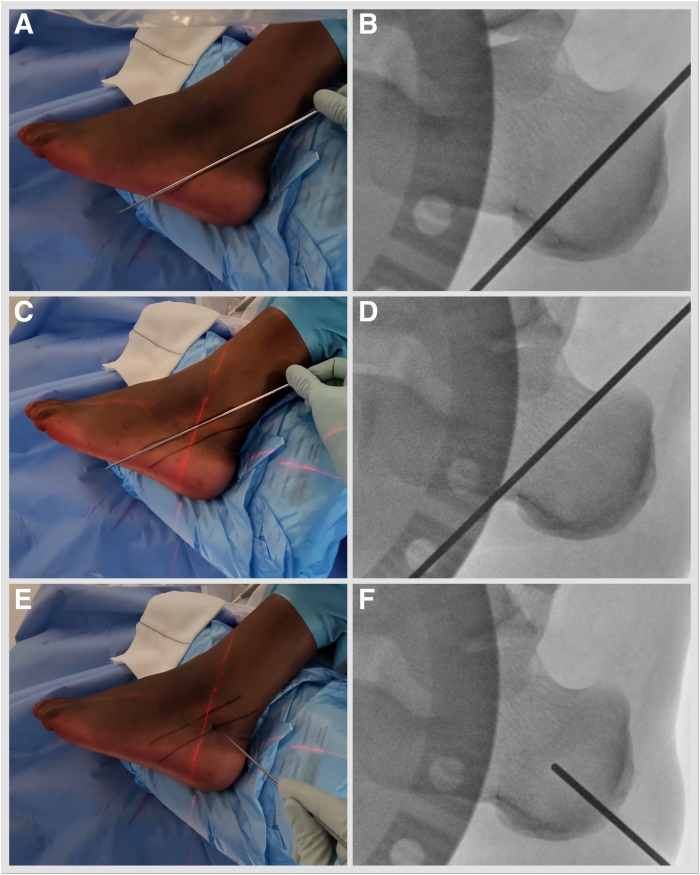
Left foot is in the lateral side-up position. Under fluoroscopy guidance, the safe zone for neural structures is marked by drawing a line joining posterior-superior and plantar prominences of the calcaneal tuberosity (A, B) and a parallel line 11 mm anterior to it (C, D). (E, F) Mark the osteotomy, starting at the midpoint of the posterior line.

**Fig 2:**
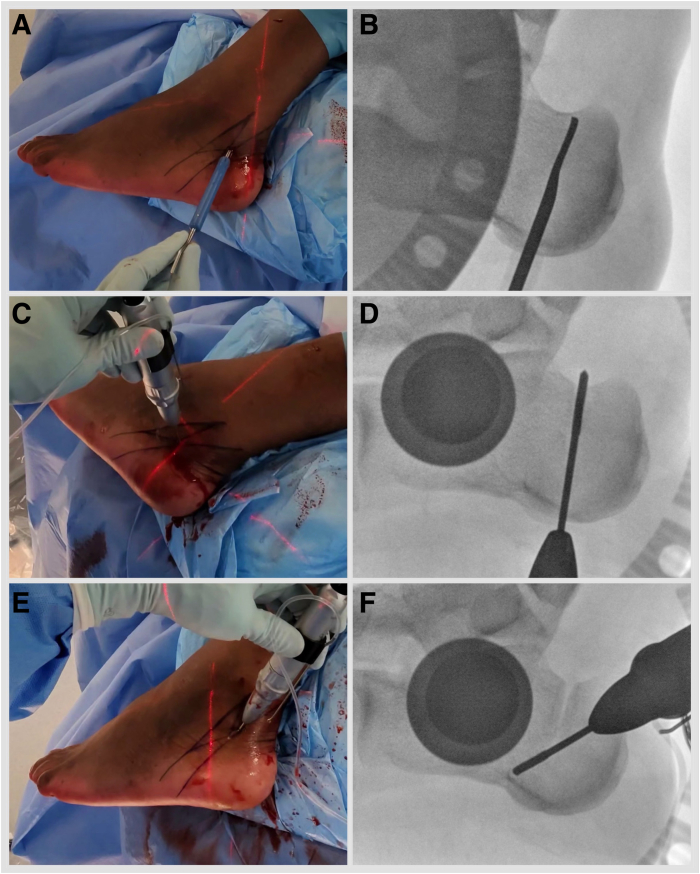
Left foot is in the lateral side-up position. (A, B) Soft tissues are elevated dorsally and plantarly off the lateral calcaneal wall using a periosteal elevator. (C, D) Using a 3 × 20-mm Shannon burr, aiming for the anterior safe zone line and the dorsal calcaneal line crossing point, proceed with a dorsal lateral quarter cut and a dorsal medial cut. (E, F) Plantar lateral quarter and plantar medial quarter cuts are parallel to the posterior safe zone line.

**Fig 3:**
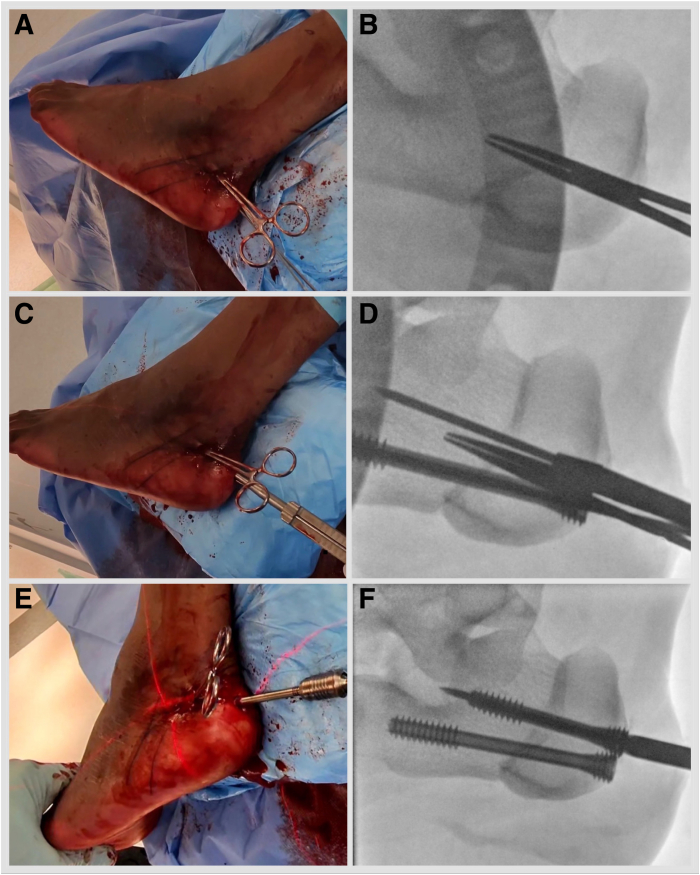
Left foot is in the lateral side-up position. (A, B) Hemostat is advanced through the osteotomy site and anterior under the lateral wall to produce a shift of 10 to 12 mm. (C) K-wires are placed: plantar wire is aimed toward the center of the anterior calcaneal process and dorsal wire short of the posterior facet subchondral bone. (D) Screw length is measured, drill holes are established, and subcortical countersinking is performed. (E, F) A shift is secured with short-thread 7-mm diameter headless compression screws.

With an appropriately anaesthetized patient in a lateral decubitus operated side-up position, a standard minimally invasive medializing calcaneal osteotomy (MIMCO) is performed. Under fluoroscopy guidance, the safe zone for neural structures is marked by drawing a line joining posterior-superior and plantar prominences of the calcaneal tuberosity and a parallel line 11 mm anterior to it.^14^ Mark the osteotomy starting point at the midpoint of the posterior line (Fig 1, Video 1). An 8- to 10-mm incision is established, and soft tissues are elevated dorsally and plantarly off the lateral calcaneal wall using a periosteal elevator. Using a 3 × 20-mm Shannon burr, aiming for the anterior safe zone line and dorsal calcaneal line crossing point, proceed with a dorsal cut. Plantar cut is parallel to the posterior safe zone line (Fig 2, Video 1). The medial cortex breech is felt by placing fingers on the medial side of the calcaneum. This technique results in a chevron cut, which is more stable but permits no rotational adjustments. Alternatively, a straight cut can be performed. A hemostat is advanced through the osteotomy site and anteriorly under the lateral wall to produce a shift of 10 to 12 mm. K-wires are placed: a plantar wire is aimed toward the center of the anterior calcaneal process and a dorsal wire short of the posterior facet subchondral bone. Screw length is measured, drill holes are established, and subcortical countersinking is performed. A shift is secured with short-thread 7-mm diameter headless compression screws (Fig 3, Video 1). Once the MIMCO is completed, the patient is positioned from lateral to supine with the foot externally rotated and over the edge of the operating table. A 4-mm 30° arthroscope, an arthroscopic 4-mm dissector, a Labral Scorpion QL (Arthrex), an arthroscopic suture passer, a No. 2 FiberLoop (Arthrex) needled loop suture, an InternalBrace Ligament Augmentation Repair Kit (Arthrex), a guide pin with eyelet, short 2.4-mm, Tenodesis Screw Master Set (Arthrex), and a fluoroscope are needed to complete the procedure.

**Fig 4:**
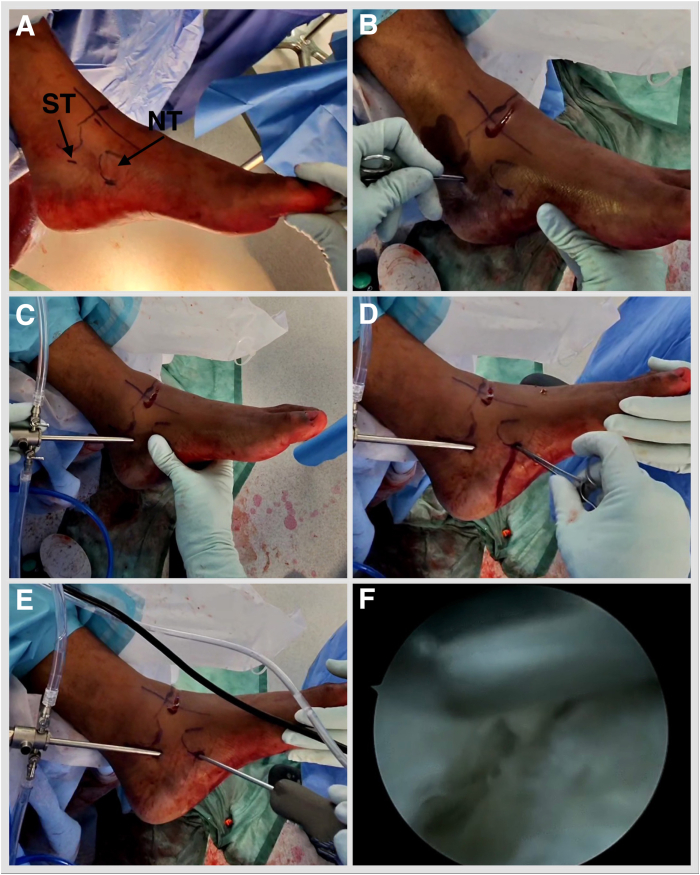
Left foot is in an externally rotated position. (A) The proximal portal is marked at the sustentaculum tali. The distal portal is marked 0.5 cm below the navicular tuberosity. (B) To establish the proximal portal, use a No. 15 scalpel blade. Perforate the investing fascia with a hemostat and direct it toward the fifth metatarsal base, then open and withdraw. (C) Arthroscope is introduced in the same trajectory. (D) Distal incision is performed, and a hemostat is advanced to view, then opened and withdrawn. (E) Shaver is introduced via the distal portal onto the arthroscope shaft and slid down to the tip of the arthroscope. (F) Shaving is commenced to clean out the soft tissue obstructing the view. (NT, navicular tuberosity; ST, sustentaculum tali.)

The operated limb is exsanguinated, and a thigh tourniquet is inflated to 100 mm Hg above the systolic blood pressure level to facilitate visibility. The proximal portal is marked at the sustentaculum tali. The distal portal is marked 0.5 cm below the navicular tuberosity (Fig 4, Video 1, Table 1). Incisions are parallel to the tendon in case conversion to an open procedure is needed. To establish the proximal portal, use a No. 15 scalpel blade and a hemostat. Perforate the investing fascia with a hemostat and direct it toward the fifth metatarsal base, then open and withdraw. An arthroscope is introduced in the same trajectory. A distal incision is performed, and a hemostat is advanced to view, then opened and withdrawn. A shaver is introduced via the distal portal onto the arthroscope shaft and slid down to the tip of the arthroscope. Shaving is commenced to clean out the soft tissue obstructing the view.

**Table 1:** Pearls and Pitfalls

Surgical Technique Steps	Pearls	Pitfalls
Marking incisions	Mark the portals before commencing the tendoscopy. Perform subtalar range of motion to identify the sustentaculum tali more easily. Palpate the tibialis posterior tendon, and mark the subtalar portal immediately inferior to it.	Failure to appropriately mark arthroscopic portals prior to tendoscopy may result in excessive portal stretching when inserting implants, risking surgical site complications.
Portals	Use the nick-and-spread technique with an intent to appreciate the pop, as the hemostat enters the investing fascia. Direct it toward the base of the fifth metatarsal before spreading and withdrawing.	Failure to appreciate the pop through the investing fascia may end up creating an unnecessary pocket superficial to the investing fascia, increasing surgical time.
FDL tendon identification	To easily identify the FDL tendon, perform deep fascia and fibrous tendon sheath dissection, plantar and medial to the posterior tibialis tendon. Stay close to the proximal portal, as the FDL is closer to the PTT proximally. Perform intermittent lesser toe range of motion for easier identification.	Failure to stay close to the proximal portal and appreciate the separate fascial layer/tendon sheath when dissecting the FDL tendon will increase surgical time and may lead to a need to convert to an open procedure. It will not compromise the surgical site if initial incisions are made parallel to the PTT.
FDL tenotomy	Ensure good tendon sheath release to allow adequate FDL delivery outside the distal portal. A hemostat is opened under the tendon to ensure the tenotomy is as distal as possible.	Failure to release the fibrous tendon sheath distally and proximally enough will decrease the ability to adequately deliver the FDL and result in a short tendon available for tenodesis.
Sustentaculum tali arm of the internal brace	Under fluoroscopy guidance, use the wire-first technique. Identify the subtalar joint and plantar slope of the sustentaculum by feeling them with a wire, confirm the starting point radiologically, and then advance the wire 15° plantar.	Failure to define the midpoint of the sustentaculum may lead to a suboptimal first anchor purchase. Failure to observe 15° of plantar angulation may result in subtalar joint penetration with the anchor.

FDL, flexor digitorum longus; PTT, posterior tibial tendon.

**Fig 5:**
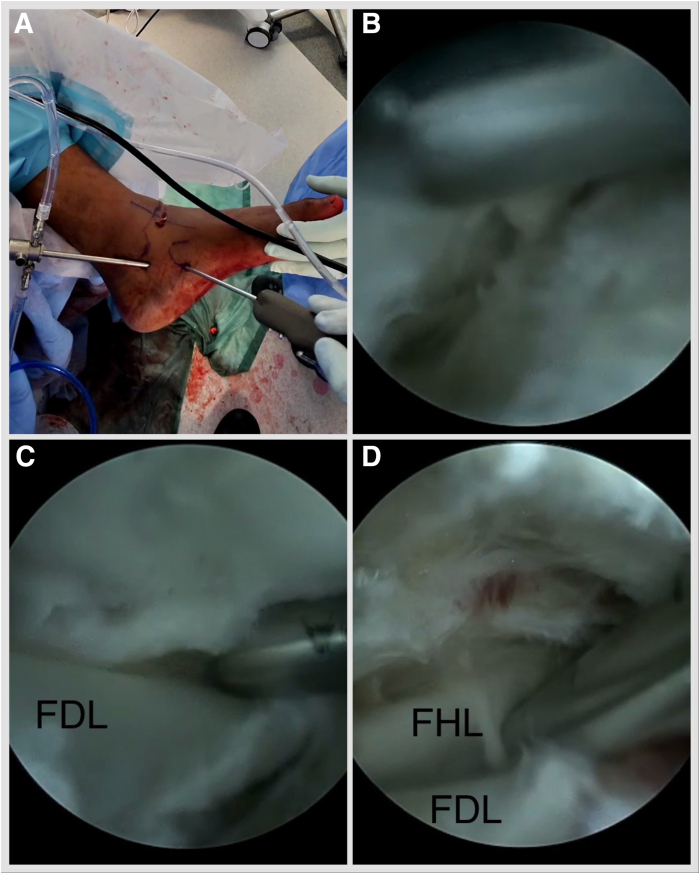
Left foot is in the externally rotated position. (A) Viewing via the proximal portal and working via the distal portal. (B) Often, the fibrous tendon sheath needs dissecting to encounter the FDL. (C) Once the FDL tendon is visualized, sheath dissection is carried out from proximal to distal until the FDL and FHL crossing point (the knot of Henry) is visualized (D). (FDL, flexor digitorum longus; FHL, flexor hallucis longus.)

**Fig 6:**
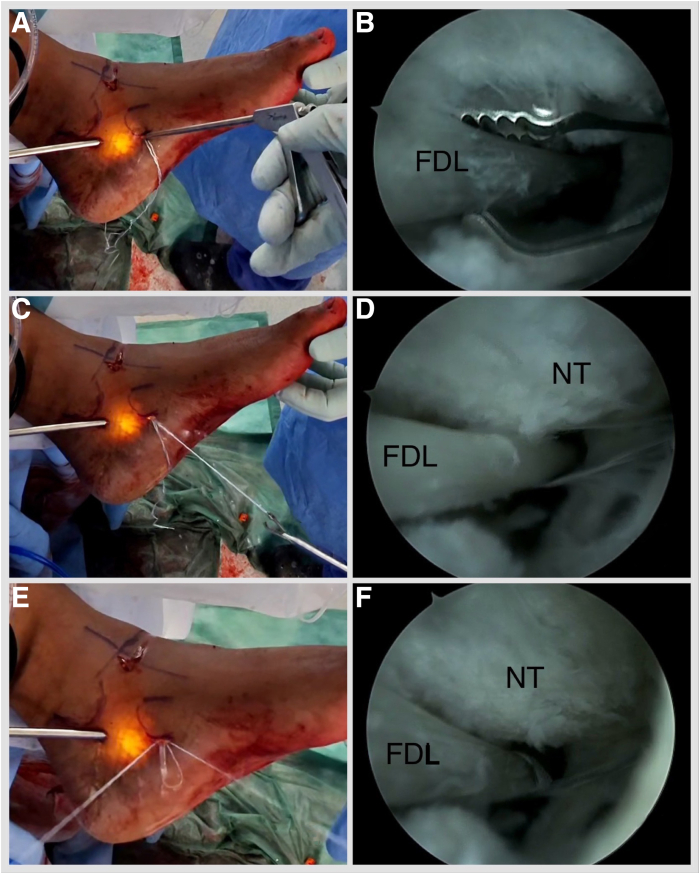
Left foot is externally rotated. (A-D) Viewing via the proximal portal and working via the distal portal, a Labral Scorpion QL (Arthrex) arthroscopic suture passer is employed to tack the FDL tendon with a FiberWire (Arthrex) suture proximal to the knot of Henry. (E, F) One strand is passed through the loop and secured by pulling the strands. (FDL, flexor digitorum longus; NT, navicular tuberosity.)

**Fig 7:**
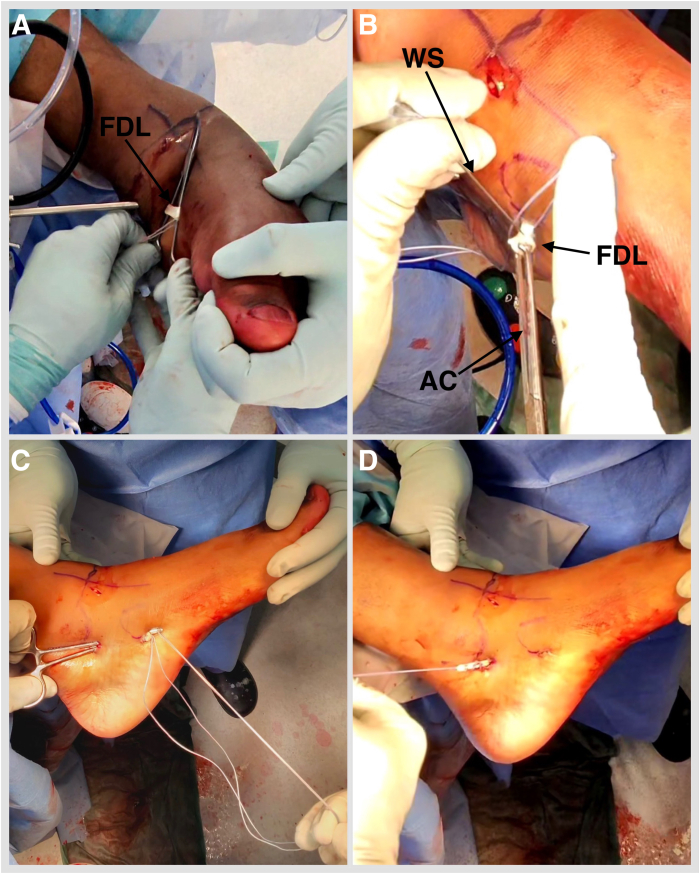
(A) Left foot is inverted, and lesser toes are plantar flexed. Deliver the FDL outside the distal portal. Place a hemostat under it and open. A tenotomy is performed at the most distal accessible point. (B) Place the Allis tissue forceps on the FDL. A retrieving stitch is removed and a No. 2 FiberLoop (Arthrex) is used to whipstitch 2.5 cm of the tendon. Retrieve the tendon through the proximal portal by placing a hemostat from proximal to distal (C) and pulling on the whipstitch (D). (AC, Allis tissue forceps; FDL, flexor digitorum longus; WS, whipstitch.)

**Fig 8:**
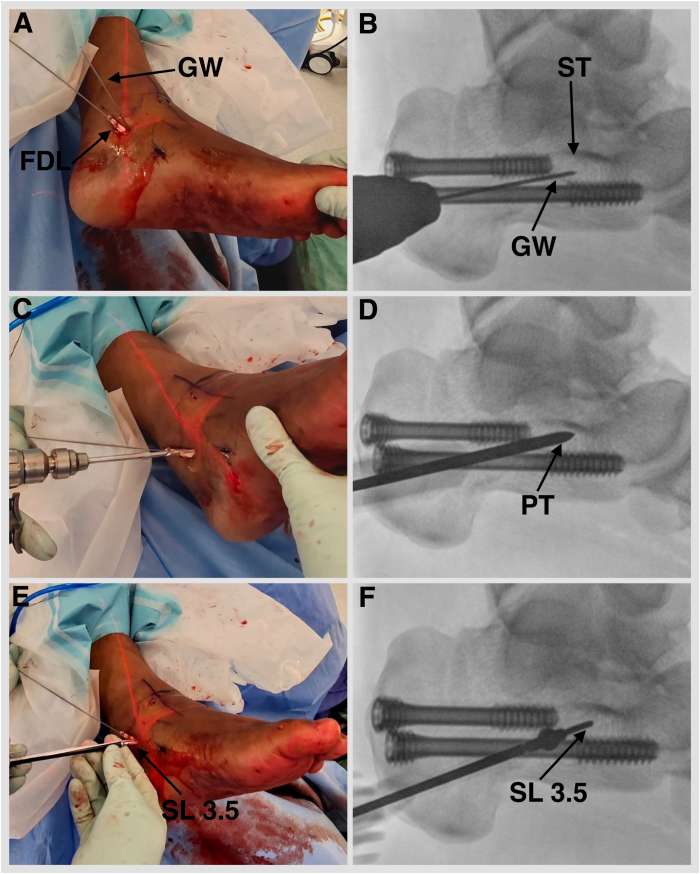
Left foot externally rotated. (A, B) Under fluoroscopy guidance, a 1.35-mm guidewire is placed into the sustentaculum tali angled 15° plantar to avoid subtalar joint penetration. (C) Drill with a cannulated 2.7-mm drill bit. (D) Tap with a 3.5-mm SwiveLock (Arthrex). (E, F) A BioComposite SwiveLock, 3.5 × 15.8 mm, loaded with a No. 2 FiberTape (Arthrex), is introduced. (FDL, flexor digitorum longus; GW, guidewire 1.35; PT, punch/tap for 3.5-mm SwiveLock; SL 3.5, SwiveLock, 3.5 × 15.8 mm; ST, sustentaculum tali.)

**Fig 9:**
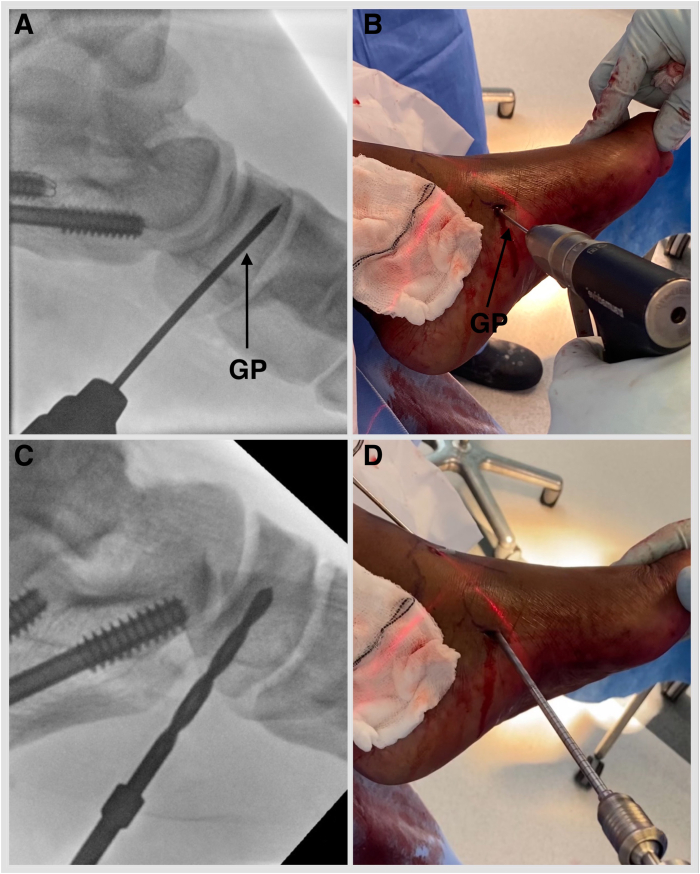
Left foot externally rotated. (A, B) Under fluoroscopy guidance, a guide pin with eyelet, measuring 2.4 mm (Beath pin; Arthrex), is introduced into the tarsal navicular from plantar medial to dorsal lateral via the distal portal. (C, D) A Cannulated Headed Reamer (Arthrex), 0.5 mm larger than the measured tendon diameter, is used to drill the navicular tunnel. (GP, guide pin with eyelet, short 2.4 mm).

**Fig 10:**
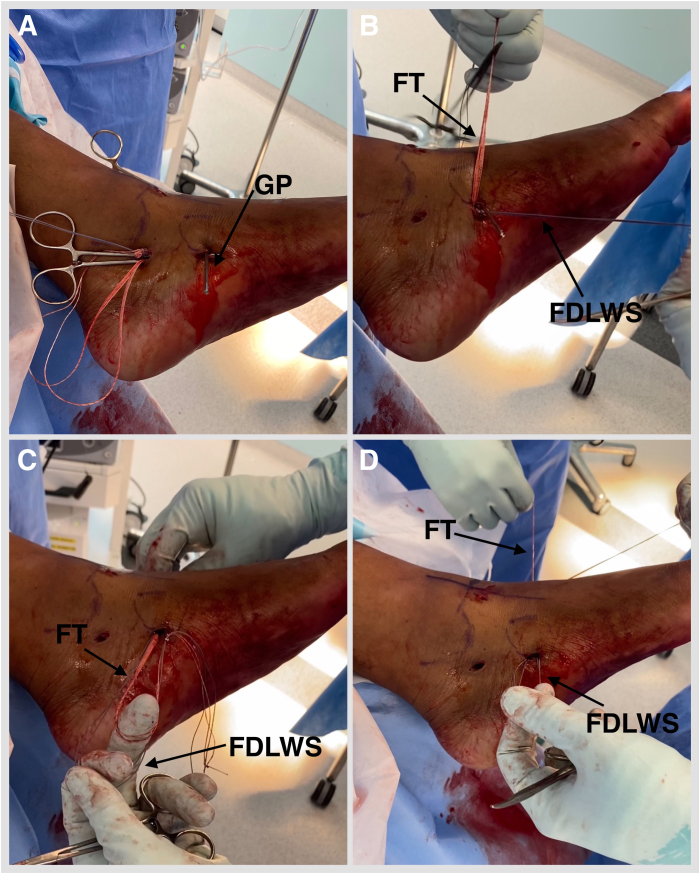
Left foot in an externally rotated position. (A, B) Retrieve the FDL tendon whipstitch and a No. 2 FiberTape from the proximal to distal portal. (C) Feed both into the guide pin with a short 2.4-mm eyelet (Arthrex). (D) Pass a no. 2 FiberTape first and then whipstitch, plantar medial to dorsal lateral, via a predrilled navicular bony tunnel. (FDLWS, flexor digitorum longus whipstitch; FT, FiberTape; GP, guide pin with eyelet.)

**Fig 11:**
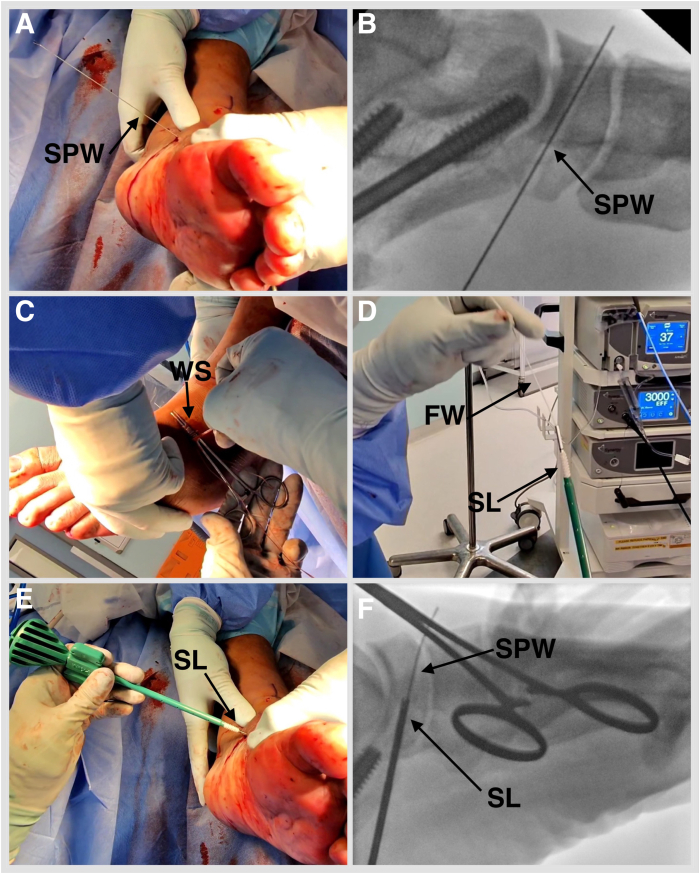
Left foot externally rotated. (A, B) Pass the flexible suture passing wire into the navicular tunnel. (C) Invert the foot, adduct the forefoot, and tighten the No. 2 FiberTape (Arthrex) by hand. Tighten the FDL by clipping and rolling the whipstitch with a hemostat on the dorsolateral side of the foot. Remove the eyelet and FiberWire from the SwiveLock, and cut the eyelet of the suture passing wire to be able to mount the SwiveLock anchor onto it. Advance the BioComposite SwiveLock, 4.75 × 19.1 mm, over the suture passing wire. (FW, FiberWire; SL, SwiveLock, 4.75 × 19.1 mm; SPW, suture passing wire; WS, whipstitch.)

**Fig 12:**
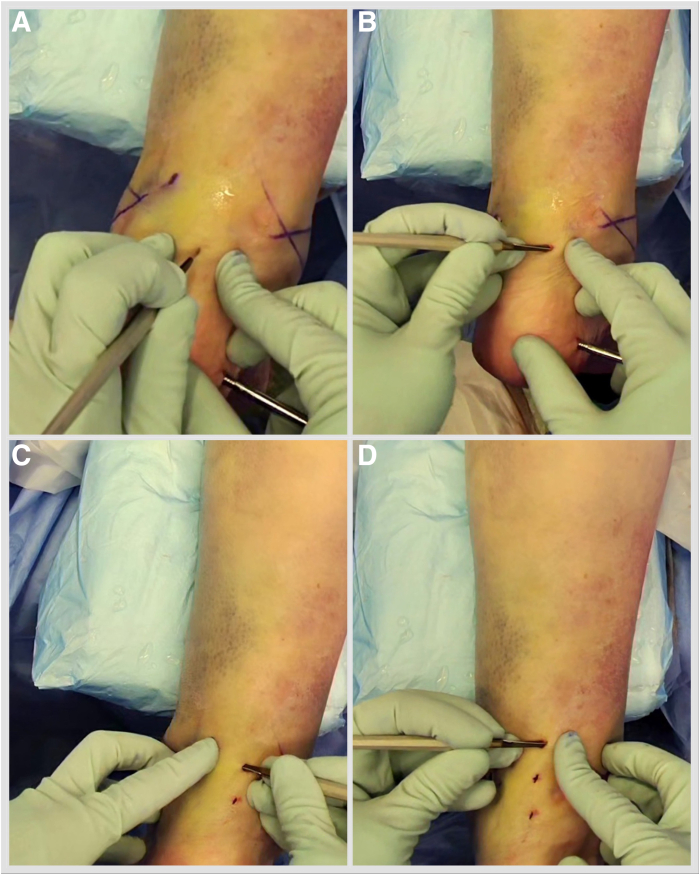
An Achilles tendon triple hemisection using the Hoke technique is performed. The foot is externally rotated by the proximal tibia to gain access to the Achilles tendon. Gentle dorsiflexion is applied to the foot. (A) One fingerbreadth proximal to the Achilles tendon insertion, advance a No. 15 scalpel blade vertically through the skin into the midline of the tendon. (B) Turn the blade 90° laterally, and complete the hemisection, feeling for completion by placing the fingers on either side of the Achilles tendon. Repeat the step, directing the hemisection medially 1 fingerbreadth proximal from the previous incision (C) and laterally 1 fingerbreadth further proximal (D).

The posterior tibial tendon can be inspected and a synovectomy performed as needed. The FDL tendon is lateral and plantar to the posterior tibial tendon. Often, the fibrous tendon sheath needs dissecting to encounter the FDL. Once the FDL tendon is visualized, sheath dissection is carried out from proximal to distal until the FDL and the flexor hallucis longus (FHL) crossing point (the knot of Henry) is visualized (Fig 5, Video 1, Table 1). FHL and FDL tendon identity is confirmed by isolated hallucal and lesser toe motion. The FDL is plantar to the FHL. The medial plantar nerve is reported to be 5.96 mm lateral to the FDL tendon at the level of the knot of Henry, and the medial plantar artery is adjacent to the tendon.^15^ A Labral Scorpion QL (Arthrex) arthroscopic suture passer is employed to tack the FDL tendon with a FiberWire (Arthrex) suture proximal to the knot of Henry. One strand is passed through the loop and secured by pulling the strands (Fig 6, Video 1). The foot is inverted, and lesser toes are plantar flexed. Deliver the FDL outside the distal portal by pulling the suture strands. Place a hemostat under it and open. A tenotomy is performed at the most distal accessible point. Place the Allis tissue forceps on the FDL. The retrieving stitch is removed, and a No. 2 FiberLoop (Arthrex) is used to whipstitch 2.5 cm of the tendon. Retrieve the tendon through the proximal portal by placing a hemostat from proximal to distal and pulling on the whipstitch (Fig 7, Video 1). Under fluoroscopy guidance, a 1.35-mm guidewire is placed into the sustentaculum tali angled 15° plantar to avoid subtalar joint penetration. Drill with a cannulated 2.7-mm drill bit, tap with a 3.5-mm SwiveLock (Arthrex), and a BioComposite SwiveLock, 3.5 × 15.8 mm, loaded with a No. 2 FiberTape (Arthrex), is introduced (Fig 8, Video 1). A guide pin with eyelet, measuring 2.4 mm (Arthrex), is introduced into the tarsal navicular from plantar medial to dorsal lateral via the distal portal. A Cannulated Headed Reamer (Arthrex), 0.5 mm larger than the measured tendon diameter, is used to drill the navicular tunnel (Fig 9, Video 1). Retrieve the FDL tendon whipstitch and a No. 2 FiberTape from the proximal to distal portal. Feed both into the eyelet of the guide pin with eyelet, measuring 2.4-mm (Arthrex) and pass a No. 2 FiberTape first and then whipstitch, plantar medial to dorsal lateral, via a predrilled navicular bony tunnel (Fig 10). Pass a flexible suture passing wire into the navicular tunnel. Invert the foot, adduct the forefoot, and tighten the No. 2 FiberTape by hand. Tighten the FDL by clipping and rolling the whipstitch with a hemostat on the dorsolateral side of the foot. Remove the eyelet and FiberWire from the SwiveLock, and cut the eyelet of the suture passing wire to be able to mount the SwiveLock anchor onto it. Advance the BioComposite SwiveLock, 4.75 × 19.1 mm, over the suture passing wire (Fig 11, Video 1). An Achilles tendon triple hemisection using the Hoke technique is performed. The foot is externally rotated by the proximal tibia to gain access to the Achilles tendon. Gentle dorsiflexion is applied to the foot. One fingerbreadth proximal to the Achilles tendon insertion, advance a No. 15 scalpel blade vertically through the skin into the midline of the tendon, turn the blade 90° laterally, and complete the hemisection, feeling for completion by placing the fingers on either side of the Achilles tendon. Repeat the step, directing the hemisection medially 1 fingerbreadth proximal from the previous incision and laterally 1 fingerbreadth further proximal (Fig 12). Incisions are closed with 3-0 Monocryl.

The back and U slab is applied for 2 weeks (Video 1). Weightbearing is fully restricted for 2 weeks. At 2 weeks, a walker boot is applied, allowing restricted weightbearing with an appropriate walking aid. At 6 weeks, weightbearing radiograms are performed. A further 4 to 6 weeks of full weightbearing with a boot are advised. Physiotherapy is commenced at 10 to 12 weeks, with an emphasis on calf and invertor strengthening. Medial arch–supporting insoles are recommended for the next 6 months.

## Discussion

Tendoscopic techniques have been known to have several advantages over open surgery, such as less pain, better cosmesis, and decreased adhesion formation (Table 2). The operative technique of posterior tibial tendon tendoscopy was first described by Wertheimer and later developed in detail by van Dijk et al. The procedure initially was limited to symptomatic vinculae resection and synovectomy in Johnson-Strom stage 1 posterior tibial tendon dysfunction.^16^, ^17^, ^18^

**Table 2:** Advantages and Disadvantages

Advantages	Disadvantages
• Potentially decreased risk of infection • Potentially decreased adhesion formation • Potentially decreased postoperative pain • Easy uncompromised conversion to open technique if needed • Aesthetically satisfactory result	• Steep but short learning curve • Possibly increased surgical time with first cases • Marginally increased cost by using a single-use Scorpion (Arthrex) needle and dissector shaver

**Fig 13:**
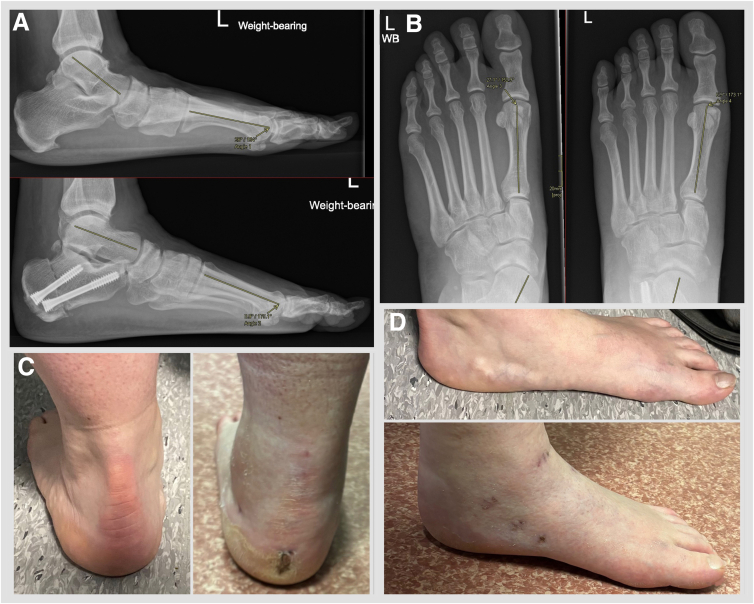
Case example. Female patient in her 50s. (A) Lateral weightbearing preoperative and 3-month postoperative radiograms show that Meary’s line apex plantar angle improved from 26° to 0.9°. (B) Dorsoplantar weightbearing preoperative and 3-month postoperative radiograms show the talus first metatarsal angle improved from 27.1° to 6.9°. (C) Clinical hindfoot preoperative and 6-week postoperative images show improved hindfoot alignment and forefoot abduction. (D) Clinical midfoot preoperative and 6-week postoperative images show improved medial longitudinal arch. Arthroscopic anterior ankle impingement decompression and anterior talofibular ligament repair were performed in this case.

Patient outcomes have not been objectively assessed for the purpose of this article; however, it is the authors’ experience that the technique is a safe procedure with a low complication profile (Fig 13).

This technique requires operating theater personnel proficient in percutaneous and endoscopic techniques. The procedure may be considered moderate to high complexity and involves a steep but short learning curve (Table 2). It can be attempted by foot and ankle surgeons trained in endoscopic techniques, including tibialis posterior tendon endoscopy.

Tendoscopic-assisted FDL tendon transfer and spring ligament synthetic suture tape reconstruction, for flexible PCFD reconstruction, is a feasible, technically demanding, but biomechanically reliable technique that addresses the etiopathological factor of the deformity procedure. In combination with MIMCO, it allows fully minimally invasive reconstruction of early-stage PCFD. Case series reporting complications, patients, and radiological outcomes are required to validate the safety and reliability of the procedure.

## Disclosures

The authors declare the following financial interests/personal relationships which may be considered as potential competing interests: D.C. reports that equipment, drugs, or supplies were provided by Arthrex GmbH, and facilities, equipment, and specimen support were provided by 10.13039/100007307Arthrex UK (Arthrex GmbH). C.N. reports that equipment, drugs, or supplies were provided by Arthrex GmbH.

## Supplementary Data

Video 1 Tendoscopic flexor digitorum longus (FDL) tendon transfer and synthetic tape spring ligament augmentation for flexible progressing collapsing foot deformity (PCFD). Procedure is indicated for patients with symptomatic flexible PCFD, with hindfoot valgus, medial longitudinal arch collapse, and forefoot abduction at the talonavicular joint up to 40°. Lateral radiograms confirm apex plantar break of Meary’s line. Dorsoplantar radiograms confirm moderate forefoot abduction. Commence with minimally invasive medializing calcaneal osteotomy. With patient in the lazy lateral position, operated leg up, and under fluoroscopy guidance, the safe zone for neural structures is marked by drawing a line joining the posterior superior and plantar prominences of the calcaneal tuberosity and a parallel line 11 mm anterior to it. Mark the osteotomy starting point at the midpoint of the posterior line. Soft tissues are elevated dorsally and plantarly off the lateral calcaneal wall using a periosteal elevator. Using a 3 × 20-mm Shannon burr, aim for the anterior safe zone line and the dorsal calcaneal line crossing point, and proceed with a dorsal cut. Plantar cut is parallel to the posterior safe zone line. A hemostat is advanced through the osteotomy site and anteriorly under the lateral wall to produce a shift of 10 to 12 mm. K-wires are placed. Plantar wire is aimed toward the center of the anterior calcaneal process and the dorsal wire short of the posterior facet subchondral bone. Screw length is measured, drill holes are established, and subcortical countersinking is performed. A shift is secured with short-thread 7-mm diameter headless compression screws. Follow with tendoscopy-assisted FDL transfer and spring ligament synthetic suture tape reconstruction. Position is supine, and the foot is externally rotated. The distal portal is marked 0.5 cm below the navicular tuberosity. The proximal portal is marked at the sustentaculum tali. Establish the proximal portal. Perforate the investing fascia with a hemostat and direct it toward the fifth metatarsal base, then open and withdraw. An arthroscope is introduced in the same trajectory. Distal incision is performed, and the hemostat is advanced to view, then opened and withdrawn. A shaver is introduced via the distal portal onto the arthroscope shaft and slid down to the tip of the arthroscope. Shaving is commenced to clean out the soft tissue obstructing the view. Often, the fibrous tendon sheath needs dissecting to encounter the FDL. Once the FDL tendon is visualized, sheath dissection is carried out from proximal to distal up to the FDL and flexor hallucis longus crossing point (the knot of Henry), which is visualized. FDL tendon’s identity is confirmed by isolated lesser toe motion. A Labral Scorpion (Arthrex) suture passer is employed to tack the FDL tendon with a FiberWire (Arthrex) suture proximal to the knot of Henry. One strand is passed through the loop and secured by pulling the strands. The foot is inverted, and the lesser toes are plantar flexed. Deliver the FDL outside the distal portal. Place a hemostat under it and open. A tenotomy is performed at the most distal accessible point. Place the Allis tissue forceps on the FDL. The retrieving stitch is removed, and a No. 2 FiberLoop (Arthrex) is used to whipstitch 2.5 cm of the tendon. Retrieve the tendon through the proximal portal by placing a hemostat from proximal to distal and pulling on the whipstitch. Under fluoroscopy guidance, a 1.35-mm guidewire is placed into the sustentaculum tali angled 15° plantar to avoid subtalar joint penetration. Drill with a cannulated 2.7-mm drill bit, tap with a 3.5-mm SwiveLock (Arthrex), and a BioComposite SwiveLock, 3.5 × 15.8 mm, loaded with a No. 2 FiberTape (Arthrex), is introduced. A guide pin with eyelet, measuring 2.4 mm (Arthrex), is introduced into the navicular bony tunnel from plantar medial to dorsal lateral via the distal portal. A Cannulated Headed Reamer (Arthrex), 0.5 mm larger than the measured tendon diameter, is used to drill the navicular tunnel. Retrieve the FDL tendon whipstitch and a No. 2 FiberTape from the proximal to distal portal. Feed both into the guide pin with a short 2.4-mm eyelet (Arthrex) and pass a No. 2 FiberTape first and then whipstitch, plantar medial to dorsal lateral, via a predrilled navicular bony tunnel. Pass a flexible suture passing wire into the navicular tunnel. Invert the foot, adduct the forefoot, and tighten the No. 2 FiberTape by hand. Tighten the FDL by clipping and rolling the whipstitch with a hemostat on the dorsolateral side of the foot. Remove the eyelet and FiberWire from the SwiveLock, and cut the eyelet of the suture passing wire to be able to mount the SwiveLock anchor onto it. Advance the BioComposite SwiveLock, 4.75 × 19.1 mm, over the suture passing wire. Final radiograms are obtained and saved on PACS. An Achilles tendon triple hemisection using the Hoke technique is performed, and incisions are closed with 3-0 Monocryl. The back and U slab is applied for 2 weeks. In the case example, at 6 weeks, Meary’s line confirms a restored medial column. At 6 weeks, the talar first metatarsal axis confirms restored forefoot abduction. At 6 weeks, weightbearing clinical images confirm restored neutral hindfoot alignment. At 6 weeks, weightbearing clinical images confirm a restored medial longitudinal arch.
